# Realization of inverse-design magnonic logic gates

**DOI:** 10.1126/sciadv.adu9032

**Published:** 2025-05-21

**Authors:** Noura Zenbaa, Fabian Majcen, Claas Abert, Florian Bruckner, Norbert J. Mauser, Thomas Schrefl, Qi Wang, Dieter Suess, Andrii V. Chumak

**Affiliations:** ^1^ Faculty of Physics, University of Vienna, Vienna 1090, Austria.; ^2^ Vienna Doctoral School in Physics, University of Vienna, Vienna 1090, Austria.; ^3^ Research Platform MMM “Mathematics-Magnetism-Materials”, University of Vienna, Vienna 1090, Austria.; ^4^ Faculty of Mathematics, University of Vienna, Vienna 1090, Austria.; ^5^ Center for Modelling and Simulation, Universität für Weiterbildung Krems, Wiener Neustadt 2700, Austria.; ^6^ School of Physics, Hubei Key Laboratory of Gravitation and Quantum Physics, Institute for Quantum, Science and Engineering, Huazhong University of Science and Technology, Wuhan, 430074, China.

## Abstract

Magnonic logic gates represent a crucial step toward realizing fully magnonic data processing systems without reliance on conventional electronic or photonic elements. Recently, a universal and reconfigurable inverse-design device has been developed, featuring a 7 by 7 array of independent current loops that create local inhomogeneous magnetic fields to scatter spin waves in an yttrium-iron-garnet film. Although initially used for linear radio frequency components, we now demonstrate key nonlinear logic gates, NOT, OR, NOR, AND, NAND, and a half-adder, sufficient for building a full processor. In this system, binary data (“0” and “1”) are encoded in the spin-wave amplitude. The contrast ratio, representing the difference between logic states, achieved values of 34, 53.9, 11.8, 19.7, 17, and 9.8 decibels for these gates, respectively.

## Teaser

Inverse design enables reconfigurable magnonic logic gates with high contrast ratios for fully magnonic data processors.

## INTRODUCTION

Logic gates are essential components in modern digital electronics, enabling complex computational tasks and serving as the backbone of modern semiconductor information processing systems. The growing demand for faster and more efficient computing has driven the development of increasingly sophisticated logic gates, which are critical for advancing computer architectures and integrated circuits. However, as traditional electronic logic gates approach their physical and thermal limits, there is increasing interest in exploring alternative technologies that can offer improved performance and efficiency. Magnonic logic gates, which use spin waves instead of charge carriers, present a promising alternative. Magnonics, the field focused on using magnons, the quanta of spin waves, for data processing and transmission, excels at promoting low-power wave computing (*1*–*4*). Spin waves operate across a wide frequency range, from the sub-gigahertz to the terahertz regime (*5*, *6*), and exhibit intrinsic nonlinear properties that can be harnessed for logic functions (*7*–*9*). For these reasons, magnetic logic gates can potentially overcome the limitations faced by conventional electronics and enable next-generation data processing technologies (*10*–*13*).

Although various approaches have successfully demonstrated magnonic logic gates, including those based on a Mach-Zehnder interferometer to realize a NOT gate (*14*), XNOR and NAND (*15*), and XOR (*16*), other approaches use dipolar coupling between magnetic waveguides to realize magnetic logic elements (*13*), like the numerically demonstrated half-adder based on two directional couplers (*17*). Spin-wave interference (*18*, *19*) and magnonic crystals (*20*) have also been used to demonstrate XNOR and magnonic analog adders and AND gates, respectively. Moreover, a recent work on complementary magnon transistors (*21*) has shown promise for the development of spin-wave processors, whereas the development of spin wave–based Ising machines (*22*) leverages spin-wave interference for solving combinatorial optimization problems. Innovations such as the SW-based 4:2 compressor using three- and five-input majority gates (*23*) further expand the computational capabilities of spin-wave logic. Last, the concept of a magnonic coprocessor (*24*) is paving the way toward an advanced type of combinatorial logic devices. Despite these advances, most designs involve time-consuming steps, often including micromagnetic simulations, followed by precise fabrication and characterization (*25*, *26*). Moreover, they are typically restricted to specific frequency ranges and perform only one functionality, limiting their flexibility.

Inverse design, an emerging concept in the field of magnonics, can be leveraged to substantially shorten the design process by automating the optimization of structures, thus reducing both time and effort while ensuring higher precision in achieving desired functionalities. This concept relies on an objective-first approach that defines a functionality and combines it with a feedback-loop optimization process. A few inverse-design magnonic functionalities have been demonstrated both numerically (*27*, *28*) and experimentally (*29*). All-optical inverse-design logic gates were recently shown only numerically in the field of photonics (*30*–*32*).

Our recent work demonstrated a reconfigurable universal inverse-design device that was used to realize multiple radio frequency (rf) components, including a reconfigurable rf filter and a frequency demultiplexer (*33*). However, all the presented results in this previous work were focused on linear functionalities, which do not apply to the implementation of binary and unconventional (neuromorphic or reservoir) computing.

Here, we present the experimental realization of inverse-design magnonic logic gates using the reconfigurable device described in (*33*). This universal device uses forward-volume magnetostatic spin-wave (FVMSW) interference to achieve constructive or destructive outputs. The design uses a 7 by 7 array of omega-shaped current loops, each generating an Oersted field of up to ±3.5 mT, to control the amplitude and phase of the spin wave propagating through inhomogeneous field regions. This creates complex interference patterns of nonlinear spin waves capable of solving versatile inverse-design problems. A feedback loop optimization iterates over the 49 current values applied to the loops, using the spin-wave signal as feedback to achieve the desired functionality. We demonstrated the device’s performance and potential by successfully implementing six key conceptual logic functions—NOT, OR, NOR, AND, NAND, and half-adder—on the same platform. Logic states “0” and “1” are encoded in spin-wave amplitudes: Signals above 90% of the maximum amplitude represent “1,” whereas those below 10% represent “0.” The device achieves high contrast ratios, up to 53.9 dB for the OR gate, demonstrating its versatility and effectiveness.

## RESULTS

### Universal inverse-design binary device

**Fig. 1.**
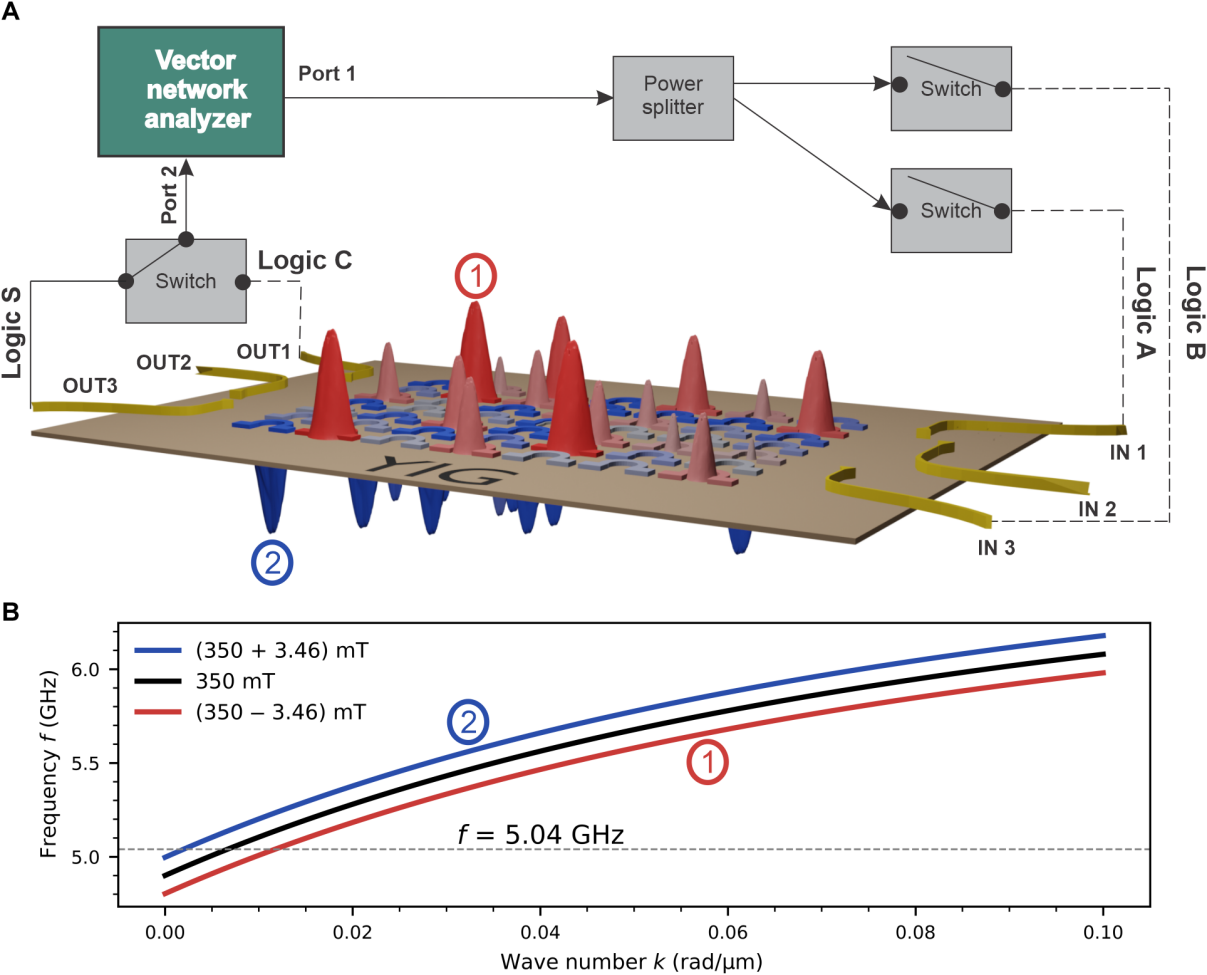
Experimental setup of the half-adder logic functionality. (**A**) Reconfigurable inverse-design device depicting the 7 by 7 omega-shaped loop array placed on top of a YIG film. It depicts the effect of the current applied to the loops as a field landscape generated locally on the film in red (film top) and blue (film bottom) for both current polarities, parallel or antiparallel to the applied out-of-plane magnetic field. It includes the setup used to realize the most complicated logic gate presented in this work, a half-adder logic functionality, which involves a VNA in a combination of a power splitter and two switches on the input side where all logic states can be achieved and one switch on the output side to measure transmission signals from both outputs. (**B**) Spin-wave dispersion relation at three different fields: 350 mT which is the external bias field fixed throughout the measurements; (350 − 3.46) mT, which is the field sensed by local spin waves at point 1 [indicated in (A)] when the Oersted field generated by a loop carrying a current of 400 mA is antiparallel to the bias field; and (350 + 3.46) mT at point 2 [indicated in (A)] when the field is generated by a loop carrying a current of the opposite polarity −400 mA and therefore parallel to the bias field. It is important to note that the maximum generated field by an omega loop does not occur at the center of the loop but around the metallic loop.

The universal inverse-design magnonic device described in (*33*) is depicted in Fig. 1A. The device is based on an yttrium-iron-garnet (YIG) film and a design region of 49 omega-shaped current loops placed on top of it to manipulate the FVMSW propagation. The omega-shaped design focuses the out-of-plane Oersted field at its center while reducing its intensity between loops. However, any configuration capable of producing a controllable, nonuniform out-of-plane magnetic field could also be effective. The current loops are controlled independently, and each can carry currents of ±1 A in 2048 steps using specially designed multichannel current sources. The current in each loop was limited to only ±400 mA in 100-mA steps to limit the device heating, which gives a total of nine discrete current values per loop. The total degrees of freedom introduced by the design region in this case are $9^{49}\approx 10^{47}$, sufficient to perform complex data processing. The physical effect of current-carrying loops is generating Oersted fields parallel or antiparallel to the applied out-of-plane external field as shown in Fig. 1A, which create local inhomogeneous magnetic field regions that force spin waves to change wavelength when propagating through them by shifting the spin-wave dispersion relations to higher or lower frequencies compared to the one at the applied bias field of 350 mT. This effect is shown in Fig. 1B for the maximum field inhomogeneity, which is ±3.46 mT at the applied current of ±400 mA. At the current setup configuration, we have no access to the characterization of the spin-wave pattern resulting from the introduced field inhomogeneity profile; further studies using Brillouin light scattering (BLS) spectroscopy or time-resolved magneto-optical Kerr effect (TR-MOKE) are needed. The device uses three input and three output transducers to cover a wide range of functionalities.

For all presented logic gates, optimization and measurement were performed at a single frequency point of 5.04 GHz. The frequency was selected based on having the same spin-wave transmission amplitude for all input-output combinations at 350 mT (see fig. S1). The measurement process starts by sending a microwave signal to the input(s), using a power splitter and mechanical switches in the case of multiple inputs. The input power is divided equally to keep the power of logic “1” the same, 25 dBm. Using the mechanical switch, the input is put to rf power “on” = “1” or rf power “off” = “0” (no applied microwave signal) to satisfy all combinations of the given truth table. In the case of a half-adder functionality, the setup used is shown in Fig. 1A. It requires two inputs A and B and two outputs C and S. For the inputs, it uses one power divider and two mechanical switches, one of which is connected to input A and the other to input B. For the state of “01,” A is “off” = “0” whereas B is “on” = “1” and vice versa for state “10.” Both A and B are set to “on” for the state “11,” and both are “off” for the state “00” (no input microwave signal sent to either). It applies the correct inputs to the corresponding state and runs the Direct Search (DS) optimization algorithm. Then, it measures two spin-wave amplitudes corresponding to outputs C and S for each input logic combination at each current set configured by the optimization algorithm via the use of another mechanical switch connected to the output transducers to switch between outputs C and S. The optimization algorithm iterates until it reaches a state that satisfies the predefined conditions. The conditions are defined as follows: In each iteration of the specific case of the half-adder, six transmission amplitudes in dB of the 5.04-GHz spin-wave frequency are recorded. These amplitudes correspond to the transmissions at output C at input states “01,” “10,” and “11,” and the other three correspond to output S at the same input states. These six transmission amplitudes are used to define the objective, where the maximum transmission of the six is considered a 100% transmission and corresponds to the output logic “1.” All other transmissions are compared to the maximum transmission value and translated into transmission ratios. The condition was unified for all logic gates presented where any output below 10% of the maximum transmission is considered to be logic “0” and all outputs above 90% of the maximum transmission are to be considered logic “1.” It is important to note that all the logic gate functionalities presented are nonlinear and therefore were realized in the spin-wave nonlinear regime at microwave power of 25 dBm (*34*).

### NOT gate

**Fig. 2.**
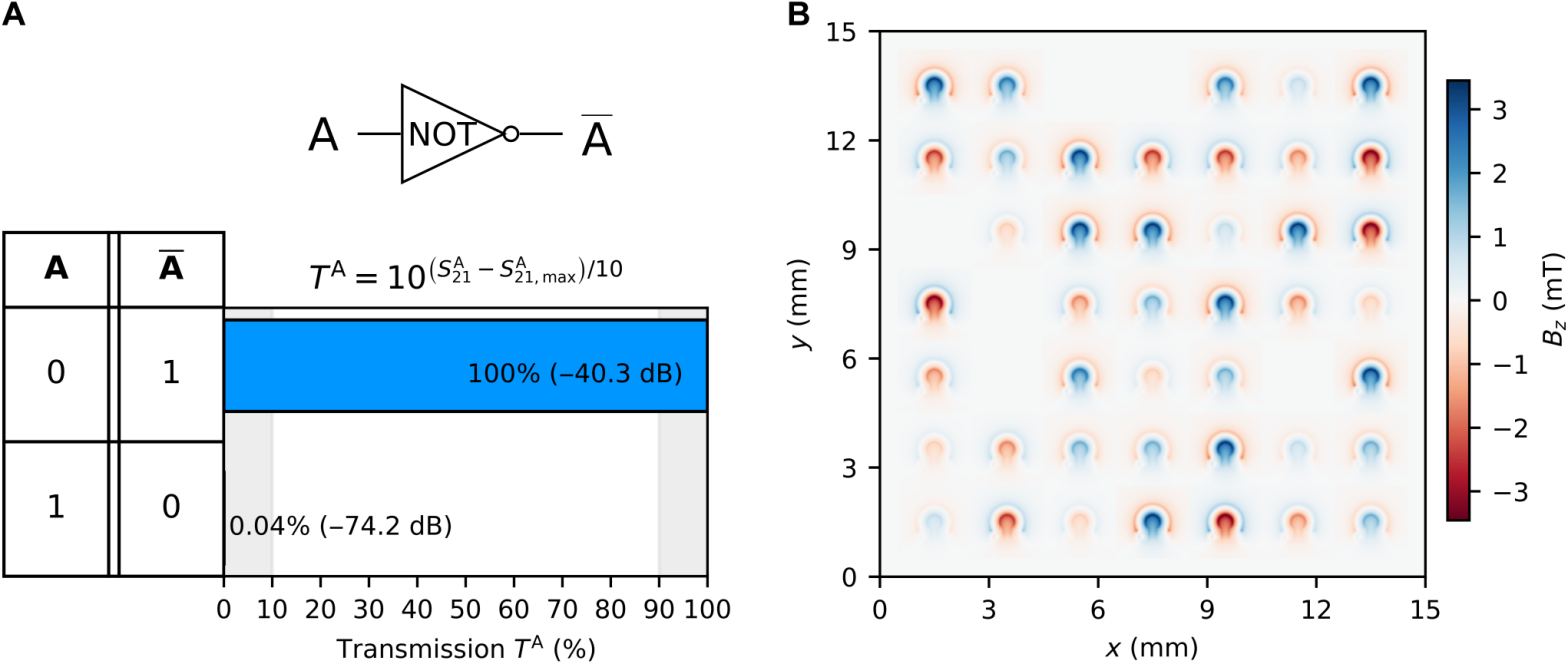
NOT logic functionality. (**A**) Spin-wave transmission percentage of output $\bar{A}$ at both logic states of input A. (**B**) Two-dimensional (2D) color map representing the Oersted field generated by the omega-shaped loops to achieve results shown in (A).

As a first example, we discuss the results of the NOT gate shown in Fig. 2. The NOT gate, or inverter, is a fundamental logic gate with one input and one output, producing the opposite logic state of its input. Input A was assigned to the IN1 transducer and output $\bar{A}$ was assigned to OUT2 (see Fig. 1A). For the gate to output a logic “1” when the input logic is “0” (no input signal), there are two possible routes. First is encoding logic states in the spin-wave phase instead of spin-wave amplitudes (*35*), and second is by using a continuous feed-line F, which is analogous to the 5-V power supply used in metal oxide semiconductor (MOS) transistors, but the feed-line uses rf current instead. The feed-line F is connected to IN3 and is always “on,” whereas input A is set to both “off” = “0” and “on” = “1” during the measurement procedure. Figure 2A shows the optimized results that represent a NOT logic function using the objective function

$$O^{\text{NOT}}=\left[T^{“0”}+\left(100-T^{“1”}\right)\right]/2$$ (1)

where $T^{“0”}$ and $T^{“1”}$ are the transmission percentages at input A logic “0” and “1,” respectively, with respect to the maximum transmission amplitude recorded per iteration. The objective function is commanded to be maximized, meaning that the closer it is to 100, the closer the optimizer is to converge and reach the functionality. The transmission percentage is calculated as follows

$$T^{A}=10^{\frac{S_{21}^{A}-S_{21,\text{max}}^{A}}{10}}\times 100$$ (2)

This calculates linear power ratios between $S_{21}^{A}$ (dB) and $S_{21,\text{max}}^{A}$ (dB), where $S_{21}^{A}$ represents the spin-wave transmission in dB from vector network analyzer (VNA) port 1 to port 2 for a given current configuration, where the input A can be either “off” = “0” or “on” = “1.” Whichever input state results in the highest transmission amplitude per iteration, $S_{21,\text{max}}^{A}$, serves as the reference for comparison with the other input. This provides a general approach for comparing relative transmission changes without assuming a specific logic gate functionality. Consequently, $S_{21,\text{max}}^{A}$ can vary across iterations. Figure 2A shows the percentage of output transmission for each given input state shown in the NOT truth table included on the left-hand side of the graph. It shows that, at the input of the “0” logic, it yields a transmission of 100% that translates to the output logic “1” and only a transmission of 0.04% at the input logic “1” that translates to the output logic “0.” In this specific case, 100% transmission in Fig. 2A corresponds to about −40 dB, which is the maximum spin-wave transmission between the input and output transducers at 5.04-GHz frequency at a specific current configuration applied, given the sample thickness, efficiency of the transducers, distance between transducers, microwave power applied, and microwave components used between the input and output. The 0.04% shown in Fig. 2A corresponds to −74 dB, which is more than 1000 times less power than the logic “1” state in this case. This results in a contrast ratio of 34 dB between the logic states “0” and “1.” The contrast ratio is defined as $S_{21}^{“1”}\left(\text{dB}\right)-S_{21}^{“0”}\left(\text{dB}\right)$, where $S_{21}^{“1”}$ represents the minimum transmission output in dB at the output logic state “1,” and $S_{21}^{“0”}$ represents the maximum transmission output in dB at the output logic state “0.” The NOT gate functionality was achieved after 295 iterations. Considering the continuous feed-line approach used in this case, this gate also does half of the functionality of an XOR gate.

The Oersted field generated by the current configuration that satisfied the NOT gate functionality is shown in a color map in Fig. 2B. Because the maximum current was limited to ±400 mA, the maximum field generated was ±3.46 mT. The current conditions are applied for all presented data.

### OR and NOR gates

**Fig. 3.**
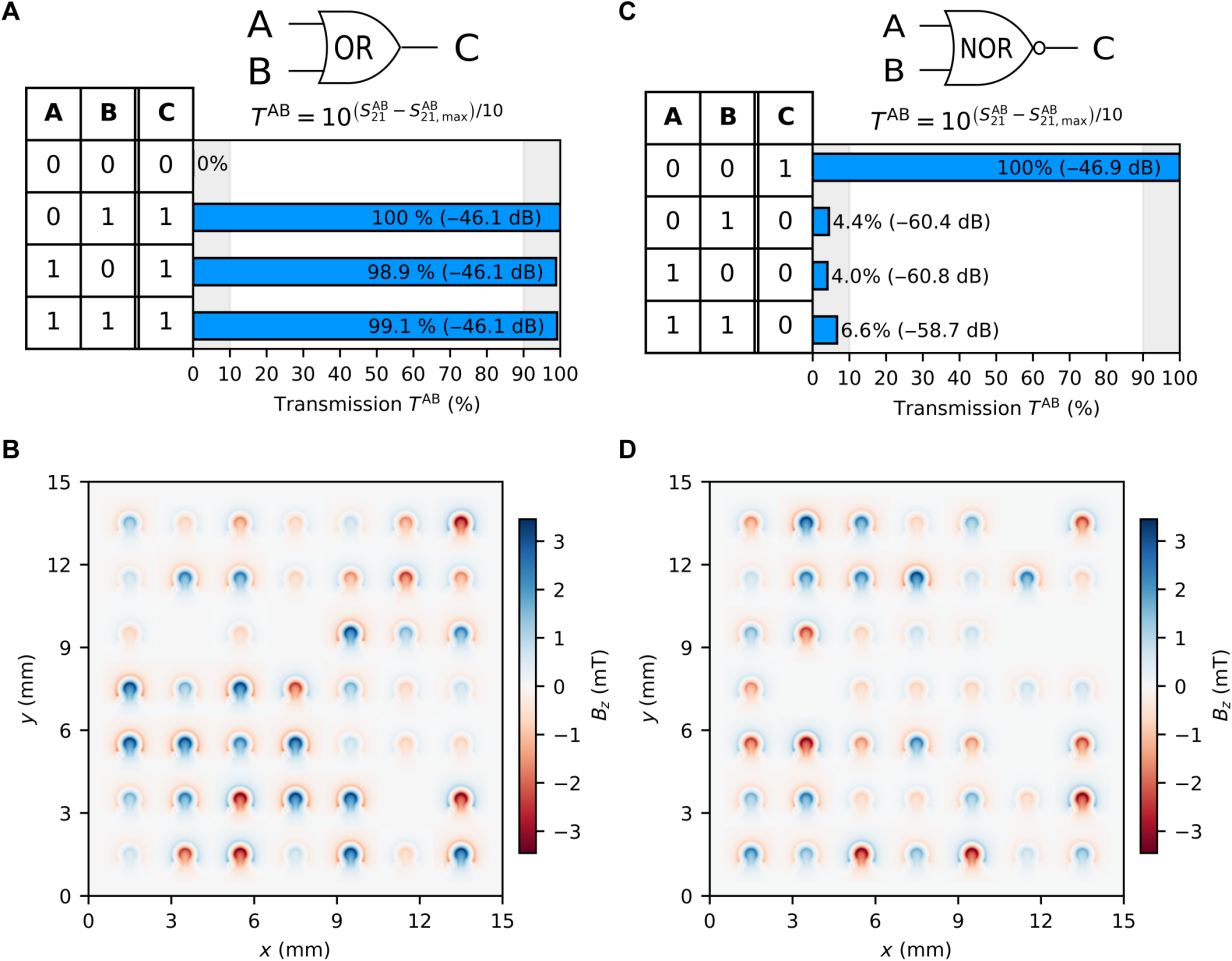
OR and NOR logic functionalities. (**A**) (OR logic gate) Spin-wave transmission percentage of output C for all logic combinations of outputs A and B. (**B**) 2D color map representing the Oersted field generated by the omega-shaped loops to achieve results shown in (A). (**C**) (NOR logic gate) Spin-wave transmission percentage of output C for all logic combinations of outputs A and B. (**D**) 2D color map representing the Oersted field generated by the omega-shaped loops to achieve results shown in (C).

Two inputs and one output are required to perform the OR logic functionality. Input A is assigned to IN1 and input B is assigned to IN3, whereas output C is assigned to OUT2 (see Fig. 1A). The optimized results of the DS algorithm are presented in Fig. 3B applying the following objective function

$$O^{\text{OR}}=\left[T^{“01”}+T^{“10”}+T^{“11”}\right]/3$$ (3)

where $T^{“01”}$, $T^{“10”}$, and $T^{“11”}$ correspond to the transmission percentages of each input state “AB.” The transmission percentages are calculated using Eq. 2. The same conditions apply, where any transmission above 90% is logic state “1” and transmissions below 10% are logic state “0.” Figure 3A shows the percentage of output transmission for each given input state shown in the OR truth table presented on the left-hand side of the graph. The OR logic gate functionality was achieved in 427 iterations. The field distribution that satisfied the 90/10 transmission condition is presented in Fig. 3B.

The NOR gate is a universal logic gate, meaning it can be combined to implement any logic function, and it outputs “0” when any input is “1,” performing the inverse of the OR gate. It relies on two inputs A and B and one output C, and they are again assigned to IN1, IN3, and OUT2 transducers. A feed-line F is needed, like in the case of the NOT gate, and is assigned to IN2. The feed-line F is always “1” during the measurements. The results of the optimization are shown in Fig. 3C and were achieved using the following objective function

$$\begin{array}{c} O^{\text{NOR}}=\left[T^{“00”}+\left(100-T^{“01”}\right)+\left(100-T^{“10”}\right)+\left(100-T^{“11”}\right)\right]/4 \end{array}$$ (4)

The NOR logic functionality was reached after 1461 iterations. Figure 3D shows the Oersted field generated distribution that satisfied the 90/10 condition for a NOR logic functionality.

### AND and NAND gates

**Fig. 4.**
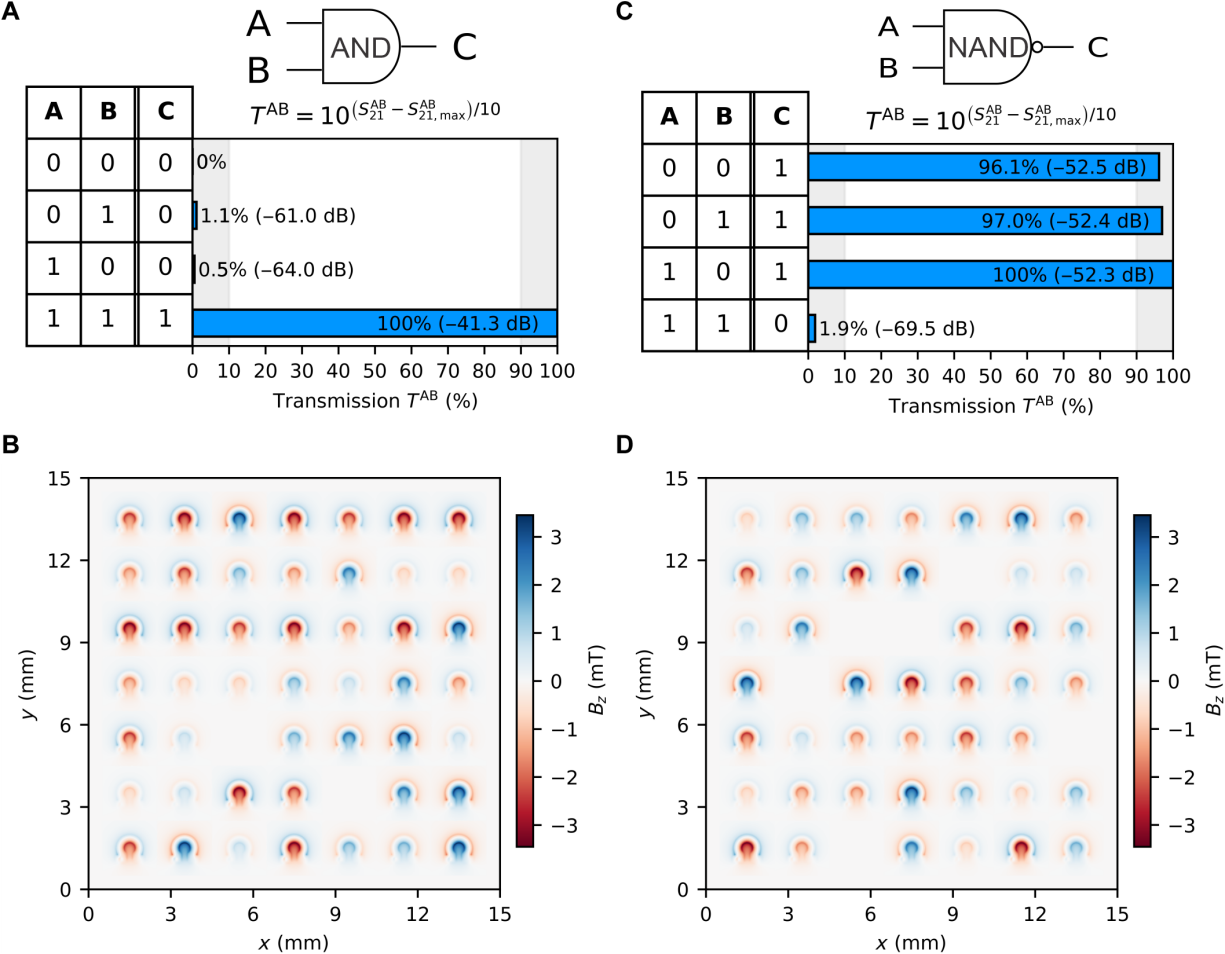
AND and NAND logic functionalities. (**A**) (AND logic gate) Spin-wave transmission percentage of output C for all logic combinations of outputs A and B. (**B**) 2D color map representing the Oersted field generated by the omega-shaped loops to achieve results shown in (A). (**C**) (NAND logic gate) Spin-wave transmission percentage of output C for all logic combinations of outputs A and B. (**D**) 2D color map representing the Oersted field generated by the omega-shaped loops to achieve results shown in (C).

The AND gate is a basic logic gate that outputs “1” only when all inputs are “1,” making it essential for requiring multiple true conditions in digital circuits. The two inputs A and B are assigned to IN1 and IN3, respectively, and output C to OUT2. The results of the DS optimization are shown in Fig. 4A using the following objective function

$$\begin{array}{c} \begin{array}{l} O^{\text{AND}}=\left[\left(100-T^{“01”}\right)+\left(100-T^{“10”}\right)+T^{“11”}\right]/3 \end{array} \end{array}$$ (5)

The AND logic gate was realized in 1118 iterations. The generated Oersted field distribution that achieved the 90/10 conditions is shown in Fig. 4B.

The NAND gate is a universal logic gate that performs the inverse of AND, outputting “0” only when all inputs are “1.” Inputs A and B are assigned to IN1 and IN3, respectively, and output C is assigned to OUT2. Similar to the NOT and NOR logic functionalities, a continuous feed-line F is used such that it is always put to “on” = “1” and is assigned to IN2. The objective function defined for the NAND gate is as follows

$$\begin{array}{c} \begin{array}{l} O^{\text{NAND}}=\left[T^{“00”}+T^{“01”}+T^{“10”}+\left(100-T^{“11”}\right)\right]/4 \end{array} \end{array}$$ (6)

The NAND logic gate functionality was reached in 803 iterations. The Oersted field distribution that achieved the 90/10 conditions is shown in Fig. 4D.

### Half-adder logic

**Fig. 5.**
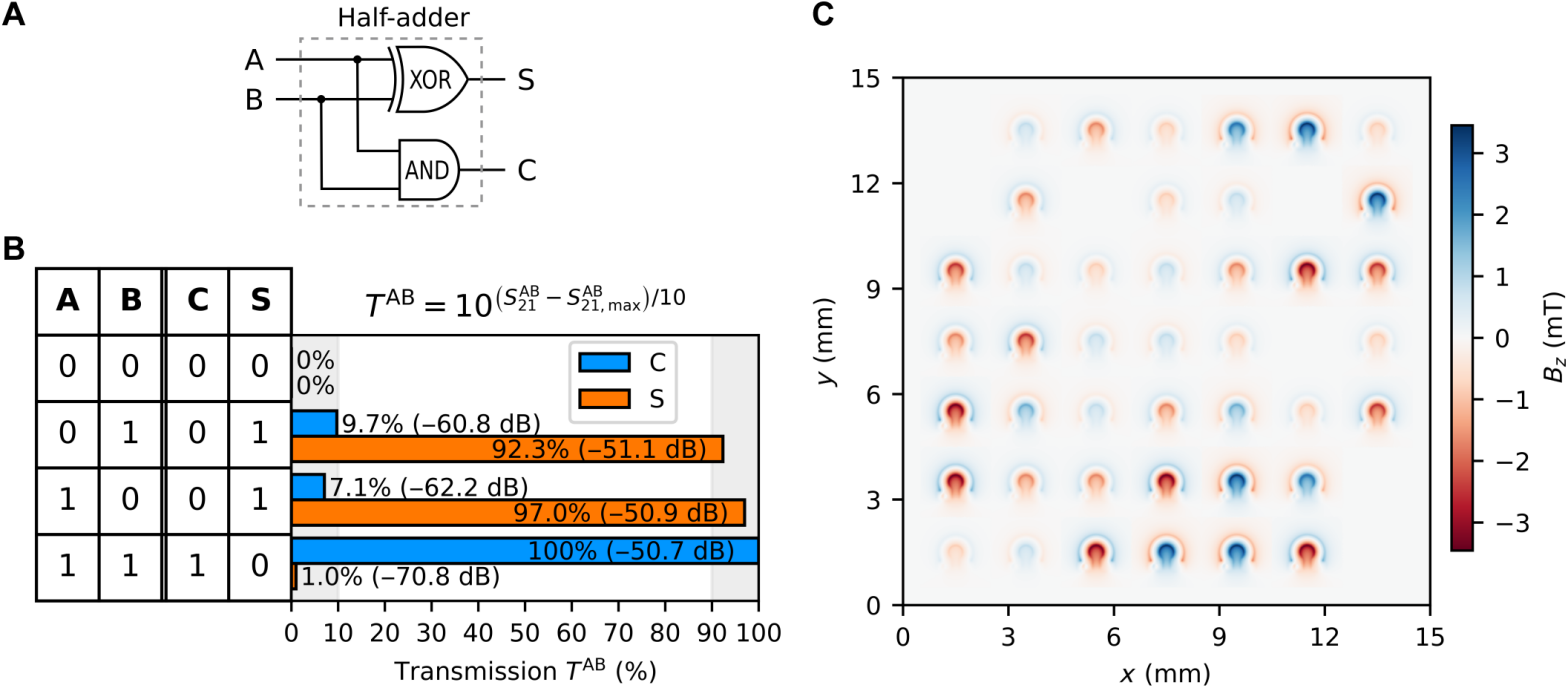
Half-adder logic functionality. (**A**) Schematic representation of half-adder gate. (**B**) Spin-wave transmission percentage of outputs S and C at all logic combinations of outputs A and B. (**C**) 2D color map representing the Oersted field generated by the omega-shaped loops to achieve results shown in (B).

A half-adder is a digital circuit that adds two binary bits, using an XOR gate for the sum and an AND gate for the carry (Fig. 5A). In our case, we did not need to implement the half-adder using separate XOR and AND gates. Instead, we achieved the truth table output of the half-adder directly with our universal device. This approach eliminates the need for a combination of specific logic gates as our universal device inherently produces the required sum and carries outputs of the half-adder truth table. Although a half-adder can handle the addition of two bits, it does not consider carrying inputs from previous operations, making it more suitable for simpler tasks or as a building block for more complex circuits like the full adder, which can manage multibit binary addition. Inputs A and B are assigned to IN1 and IN3, respectively, whereas outputs C and S are assigned to OUT1 and OUT3, respectively. The objective function defined to be maximized by the DS optimizer is as follows

$$\begin{array}{c} \begin{array}{l} O^{\text{half-}\text{adder}}=\left[\left(100-T_{C}^{“01”}\right)+T_{S}^{“01”}+\left(100-T_{C}^{“10”}\right)\right.\\ \left.\;,+T_{S}^{“10”}+T_{C}^{“11”}+\left(100-T_{S}^{“11”}\right)\right]/6 \end{array} \end{array}$$ (7)

where the subscripts (C) and (S) correspond to the two outputs C and S, respectively. The transmission percentages for both outputs are given in Fig. 5B. The half-adder logic functionality needed 653 iterations to be realized. The color map of the field distribution generated by the omega-shaped loop array to realize the inverse-design half-adder logic functionality is shown in Fig. 5C.

## DISCUSSION

Although spin waves offer inherent advantages such as ultralow-power operation and high-frequency signal processing, their practical implementation requires addressing key challenges related to power consumption, scalability, and speed. The present implementation operates at 25-dBm input power, but at the nanoscale, nonlinear effects become much more pronounced in confined magnetic volumes, enabling spin-wave generation at much lower applied powers. Previous studies (*34*, *36*) have demonstrated that nonlinear spin waves can be excited efficiently at reduced power levels that can reach down to 6 dBm. In addition, localizing rf excitation within targeted regions could further minimize energy requirements, making the system more energy-efficient and adaptable for integrated applications.

Beyond power reduction, a key step toward scalability is replacing the current loops, which introduce additional ohmic losses, with a nonvolatile approach. Instead of relying on continuous electrical currents, field inhomogeneities could be generated using individually controlled nanomagnets of complex shapes, a method well studied in the field of magnonics (*37*–*39*). This approach would provide multiple degrees of freedom in spin-wave control while greatly reducing energy dissipation, as the system would consume energy only during reconfiguration and remain passive during operation. Transitioning to such a design would pave the way toward more practical, scalable spin wave–based computing architectures. Future work will focus on refining these strategies, optimizing energy efficiency, and exploring advanced excitation mechanisms that align with emerging low-power computing paradigms.

In terms of energy consumption, a relevant frame of reference is the in-plane magnetized half-adder demonstrated in (*17*), which consumes only about 2 aJ per operation. However, in the forward-volume configuration, spin-wave nonlinearity effects are much more pronounced (*34*), suggesting that energy consumption could be even lower in our approach. In addition, considering the device density, we consider the most ideal case of the nonvolatile approach proposed above of using simple nanomagnets with two discrete states, instead of the dc loops, that would require the use of ~150 nanomagnets to match the degrees of freedom introduced in our current design. Given that each nanomagnet has dimensions of 10 nm by 10 nm, they could be arranged in a 10 by 15 array and occupy a total area of 100 nm by 150 nm, or 0.015 μm^2^. This footprint is ~68 times smaller than that of a CMOS (complementary metal-oxide semiconductor)–based half-adder (*17*), highlighting the potential advantages of spin-wave computing in high-density logic applications.

As we explore practical implementation, the data rate represents another key parameter for further improving system performance. Although we focused on single-frequency operation in this study to maximize optimization speed, our previous work (*33*) demonstrated that stop bands with a width of at least 10 MHz can be realized, enabling operation with pulse durations as short as 100 ns. Further increasing the clock speed should be feasible but will require dedicated studies.

In addition, meeting the fundamental preconditions for digital logic is essential for practical computation. Our implementation already satisfies the requirements of nonlinearity and a complete set of gates. Regarding cascading of logic gates (precondition 3), we demonstrated a half-adder where XOR and AND functionalities are realized within a single device, eliminating the need for cascading separate gates. A similar approach could be extended to more complex logic operations, such as a full adder or a majority gate. The same approach was used to demonstrate theoretically a 32-bit ripple carry adder (*26*). However, cascading multiple gates will require a spin-wave amplifier, an area that remains under active investigation. Input-output isolation (precondition 4) has been explored in a theoretical study on nonreciprocal inverse-designed Y-circulators (*27*), where the objective function was optimized to minimize backflow from the output to input. Although this principle is promising, further experimental validation is needed to confirm its feasibility. Last, achieving power gain (precondition 5) will necessitate a spin-wave amplifier or an electromagnetic amplifier after the microwave transducer, both of which remain ongoing research topics. Addressing these aspects systematically will be crucial for advancing spin-wave computing toward practical, scalable digital architectures.

In summary, we presented the inverse design of all-magnonic logic gates, including NOT, OR, NOR, AND, NAND, and half-adder logic functionalities. They were all realized at a spin-wave frequency of 5.04 GHz and microwave power of 25 dBm. By introducing a feed-line F, we made it possible to achieve a logic state “1” at the output for operations like “0 NOT = 1” and “0 NOR 0 = 1.” The optimization procedure used is the DS algorithm. The contrast ratios achieved are 34, 53.9, 11.8, 19.7, 17, and 9.8 dB for the NOT, OR, NOR, AND, NAND, and half-adder, respectively.

These results demonstrate the feasibility of inverse-designed spin-wave logic gates using nonlinear spin-wave physics and highlight key considerations for scalability and practical implementation. Future advancements will focus on reducing power consumption by leveraging nonlinear spin-wave effects in forward-volume configurations in the nanoscale, as well as transitioning to a nonvolatile approach using nanomagnets to enhance energy efficiency. In addition, optimizing spin-wave amplification and ensuring input-output isolation will be crucial for enabling large-scale cascading of logic operations.

## MATERIALS AND METHODS

### Experimental setup

#### YIG sample

The universal device is based on an 18-μm–thick YIG rectangular film with dimensions of 24 mm by 17.5 mm, grown using liquid phase epitaxy (LPE) on a 500-μm–thick gadolinium-gallium-garnet (GGG) substrate (*40*). LPE is a growth technique that ensures that the YIG film and GGG substrate are lattice matched, reducing spin-wave damping by promoting optimal crystal alignment.

#### Design region

The 15 mm–by–15 mm design region is composed of 49 omega-shaped loops and is printed on a printed circuit board (PCB). The width of one loop is about 1.1 mm with 2-mm distance between adjacent loops. The PCB consists of four metallic layers, with the design region placed on the top layer, placed on the YIG film. To avoid spin-wave scattering caused by the metallic loops, a Teflon layer was used between the YIG and the PCB. It also served as a heat insulator to prevent heat transfer from the loops to the YIG sample.

#### Independent control of current loops

Five multichannel current sources, developed by ElbaTech Srl, with 10 channels each, were used. They were controlled via a PC and designed with feedback loops, enabling precise independent current application in the range of ±1 A in 2048 steps for each channel.

#### Microwave transducers

Copper microstrip transmission lines, fabricated on a duroid substrate, served as microwave transducers. These lines are designed with 50-ohm impedance at their base and taper down to a width of 50 μm, allowing the excitation of spin waves over a broad wave number range, from 3.55 rad/cm to 0.111 rad/μm (*41*). The distance traveled by the waves from input to output transducers is about 2.2 cm.

#### Bias magnetic field

An external magnetic field was applied perpendicular to the sample plane using an electromagnet, with the field strength held at 350 mT.The field was regulated by a custom-built magnet driver, which continuously monitored and adjusted the field during the experiment, ensuring precision down to ±0.012 mT. Water-cooled magnet poles were used to dissipate excess heat generated by the PCB carrying the current loops.

#### Vector network analyzer

To excite and detect spin waves, the setup used a two-port VNA in conjunction with multiple mechanical switches, allowing signals to be monitored on both the input and output sides of the device. The VNA supported frequency measurements from 10 MHz to 20 GHz. All data collection and experimental algorithms were implemented using Python libraries.

### DS algorithm

All presented logic gates were optimized using a DS optimization process explained in (*33*). The DS algorithm, conceptually similar to the direct binary search (DBS) algorithms used in (*27*, *42*), differs by using a predefined set of finite values, rather than a binary scheme, to identify the optimal solution. The process starts by generating an initial random configuration of currents, denoted as $I_{0}\left(n\right)$, where n represents the number of current loop. This initial configuration consists of 49 current loops, each assigned a random value from the set $S=\left(i_{1},i_{2},.\ldots ,i_{k}\right)$. The algorithm then evaluates the objective function O for the initial state and proceeds by selecting a random loop n, checking its current value (e.g., $i_{j}$), and randomly selecting a different value from the set $S–\left\{i_{j}\right\}$. After updating the current, it recalculates the objective function and compares it to the original O. Depending on which value yields a higher objective, it either retains the previous current or applies the new one. This process continues sequentially through all 49 loops, modifying each loop one by one. Once all loops have been altered in this manner, the first iteration is complete, resulting in the updated configuration $I_{1}\left(n\right)$. One of the disadvantages of using this optimization algorithm is that it does not introduce enough randomness to find global maxima and can easily get stuck in a local maximum. This was solved by allowing the algorithm to start from a new random state once it gets stuck in a local maximum until it reaches a maximum that satisfies the condition imposed by the objective function.

## Supplementary Materials

### This PDF file includes:

Supplementary Text Fig. S1 References
