# Supplementary material for: Realization of inverse-design magnonic logic gates
Source: Sci Adv. 2025 May 21;11(21):eadu9032. doi: 10.1126/sciadv.adu9032 (PMC12094216; doi:10.1126/sciadv.adu9032)
Supplement: Supplementary file 1 — Supplementary Text Fig. S1 References [file sciadv.adu9032_sm.pdf]

Science Advances
AAAS

Supplementary Materials for

Realization of inverse-design magnonic logic gates

Noura Zenbaa *et al.*

Corresponding author: Noura Zenbaa, noura.zenbaa@univie.ac.at; Andrii V. Chumak, andrii.chumak@univie.ac.at

Sci. Adv. **11**, eadu9032 (2025)
DOI: 10.1126/sciadv.adu9032

This PDF file includes:

Supplementary Text
Fig. S1
References

Spin-wave full transmission spectrum at 350 mT

Figure S1A and S1B show the full signal of the spin-wave transmission at 350 mT. They show the reference signal (at zero applied currents in the omega-shaped loops) of all the input-output combinations of the most complex functionality of a half-adder. Inputs A and B are assigned to IN1 and IN3, respectively, while outputs C and S are assigned to OUT1 and OUT3, respectively. The four spectra show insertion losses that range from -30 dB to -40 dB at the working frequency of 5.04 GHz, which means that the maximum transmission of the binary state "1" will not exceed this range. These insertion losses can be reduced by improving spin-wave excitation and detection efficiency through proper transducer design, coupled with reducing the transducer separation through a more compact design. Through these improvements, insertion losses as low as -2.5 dB can be achieved (*43, 44*).

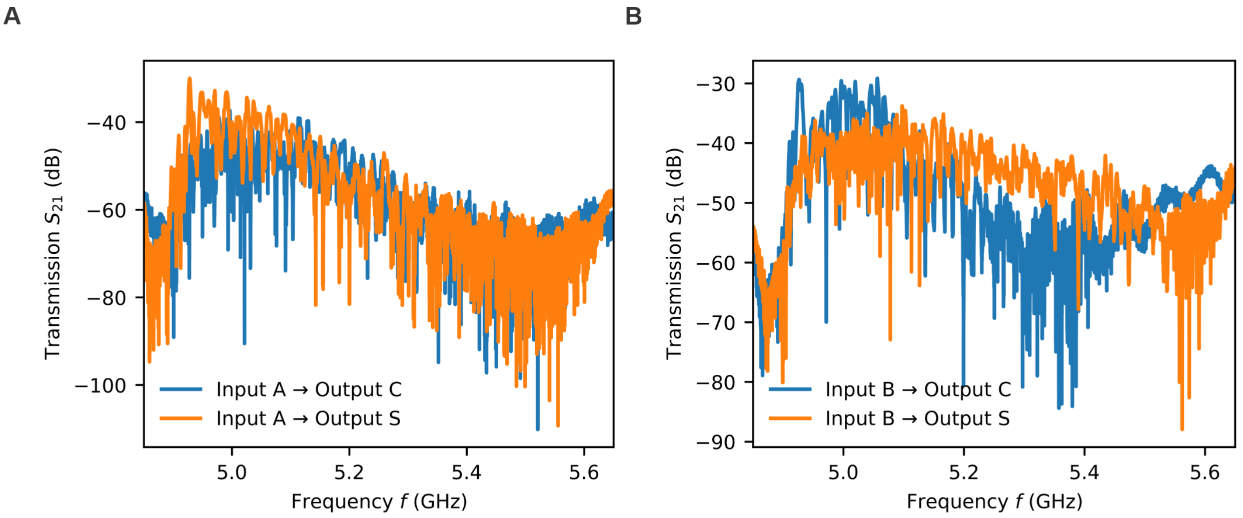

Figure S1: **Transmission spectra of half-adder at zero-current applied**. **A** Two transmission spectra from input A to both outputs C and S. **B** Two transmission spectra from input B to both outputs C and S.

REFERENCES AND NOTES

1. A. Barman, G. Gubbiotti, S. Ladak, A. O. Adeyeye, M. Krawczyk, J. Gräfe, C. Adelmann, S. Cotofana, A. Naeemi, V. I. Vasyuchka, B. Hillebrands, S. A. Nikitov, H. Yu, D. Grundler, A. V. Sadovnikov, A. A. Grachev, S. E. Sheshukova, J.-Y. Duquesne, M. Marangolo, G. Csaba, W. Porod, V. E. Demidov, S. Urazhdin, S. O. Demokritov, E. Albisetti, D. Petti, R. Bertacco, H. Schultheiss, V. V. Kruglyak, V. D. Poimanov, S. Sahoo, J. Sinha, H. Yang, M. Münzenberg, T. Moriyama, S. Mizukami, P. Landeros, R. A. Gallardo, G. Carlotti, J.-V. Kim, R. L. Stamps, R. E. Camley, B. Rana, Y. Otani, W. Yu, T. Yu, G. E. W. Bauer, C. Back, G. S. Uhrig, O. V. Dobrovolskiy, B. Budinska, H. Qin, S. v. Dijken, A. V. Chumak, A. Khitun, D. E. Nikonov, I. A. Young, B. W. Zingsem, M. Winklhofer, The 2021 magnonics roadmap. *J. Phys. Condens. Matter* **33**, 413001 (2021).

2. M. Iwaba, K. Sekiguchi, Spin-wave switching using dynamic magnonic crystal. *Appl. Phys. Express* **14**, 073002 (2021).

3. A. V. Chumak, P. Kabos, M. Wu, C. Abert, C. Adelmann, A. O. Adeyeye, J. Åkerman, F. G. Aliev, A. Anane, A. Awad, C. H. Back, A. Barman, G. E. W. Bauer, M. Becherer, E. N. Beginin, V. A. S. V. Bittencourt, Y. M. Blanter, P. Bortolotti, I. Boventer, D. A. Bozhko, S. A. Bunyaev, J. J. Carmiggelt, R. R. Cheenikundil, F. Ciubotaru, S. Cotofana, G. Csaba, O. V. Dobrovolskiy, C. Dubs, M. Elyasi, K. G. Fripp, H. Fulara, I. A. Golovchanskiy, C. Gonzalez-Ballestero, P. Graczyk, D. Grundler, P. Gruszecki, G. Gubbiotti, K. Guslienko, A. Haldar, S. Hamdioui, R. Hertel, B. Hillebrands, T. Hioki, A. Houshang, C.-M. Hu, H. Huebl, M. Huth, E. Iacocca, M. B. Jungfleisch, G. N. Kakazei, A. Khitun, R. Khymyn, T. Kikkawa, M. Kläui, O. Klein, J. W. Kłos, S. Knauer, S. Koraltan, M. Kostylev, M. Krawczyk, I. N. Krivorotov, V. V. Kruglyak, D. Lachance-Quirion, S. Ladak, R. Lebrun, Y. Li, M. Lindner, R. Macêdo, S. Mayr, G. A. Melkov, S. Mieszczak, Y. Nakamura, H. T. Nembach, A. A. Nikitin, S. A. Nikitov, V. Novosad, J. A. Otálora, Y. Otani, A. Papp, B. Pigeau, P. Pirro, W. Porod, F. Porrati, H. Qin, B. Rana, T. Reimann, F. Riente, O. Romero-Isart, A. Ross, A. V. Sadovnikov, A. R. Safin, E. Saitoh, G. Schmidt, H. Schultheiss, K. Schultheiss, A. A. Serga, S. Sharma, J. M. Shaw, D. Suess, O. Surzhenko, K. Szulc, T. Taniguchi, M. Urbánek, K. Usami, A. B. Ustinov, T. van der Sar, S. van Dijken, V. I. Vasyuchka, R. Verba, S. V. Kusminskiy, Q. Wang, M. Weides, M. Weiler, S.

Wintz, S. P. Wolski, X. Zhang, Advances in magnetics roadmap on spin-wave computing. *IEEE Trans. Magn.* **58**, 1–72 (2022).

4. Y. V. Khivintsev, V. K. Sakharov, A. V. Kozhevnikov, G. M. Dudko, Y. A. Filimonov, A. Khitun, Spin waves in YIG based magnonic networks: Design and technological aspects. *J. Magn. Magn. Mater.* **545**, 168754 (2022).

5. T. Kampfrath, A. Sell, G. Klatt, A. Pashkin, S. Mährlein, T. Dekorsy, M. Wolf, M. Fiebig, A. Leitenstorfer, R. Huber, Coherent terahertz control of antiferromagnetic spin waves. *Nat. Photonics* **5**, 31–34 (2011).

6. Y. Wu, M. Elyasi, X. Qiu, M. Chen, Y. Liu, L. Ke, H. Yang, High-performance THz emitters based on ferromagnetic/nonmagnetic heterostructures. *Adv. Mater.* **29**, 1603031 (2017).

7. P. Krivosik, C. E. Patton, Hamiltonian formulation of nonlinear spin-wave dynamics: Theory and applications. *Phys. Rev. B* **82**, 184428 (2010).

8. A. V. Chumak, A. A. Serga, B. Hillebrands, Magnon transistor for all-magnon data processing. *Nat. Commun.* **5**, 4700 (2014).

9. A. V. Sadovnikov, E. N. Beginin, M. A. Morozova, Y. P. Sharaevskii, S. V. Grishin, S. E. Sheshukova, S. A. Nikitov, Nonlinear spin wave coupling in adjacent magnonic crystals. *Appl. Phys. Lett.* **109**, 042407 (2016).

10. A. Khitun, M. Bao, K. L. Wang, Magnonic logic circuits. *J. Phys. D Appl. Phys.* **43**, 264005 (2010).

11. G. Talmelli, T. Devolder, N. Träger, J. Förster, S. Wintz, M. Weigand, H. Stoll, M. Heyns, G. Schütz, I. P. Radu, J. Gräfe, F. Ciubotaru, C. Adelmann, Reconfigurable submicrometer spin-wave majority gate with electrical transducers. *Sci. Adv.* **6**, eabb4042 (2020).

12. C. Adelmann, F. Ciubotaru, F. Meng, S. Cotofana, S. Couet, "Spintronic logic: From transducers to logic gates and circuits," in *2023 IEEE International Magnetic Conference - Short Papers (INTERMAG Short Papers)* (IEEE, 2023), pp. 1–2 .

13. R. Hayashi, S. Nezu, K. Sekiguchi, Enhanced signal-to-noise ratio in magnonic logic gates via dipole coupling. *Phys. Rev. Appl.* **22**, 034037 (2024).

14. M. P. Kostylev, A. A. Serga, T. Schneider, B. Leven, B. Hillebrands, Spin-wave logical gates. *Appl. Phys. Lett.* **87**, 153501 (2005).

15. T. Schneider, A. A. Serga, B. Leven, B. Hillebrands, R. L. Stamps, M. P. Kostylev, Realization of spin-wave logic gates. *Appl. Phys. Lett.* **92**, 022505 (2008).

16. A. B. Ustinov, E. Lähderanta, M. Inoue, B. A. Kalinikos, Nonlinear spin-wave logic gates. *IEEE Magn. Lett.* **10**, 1–4 (2019).

17. Q. Wang, M. Kewenig, M. Schneider, R. Verba, F. Kohl, B. Heinz, M. Geilen, M. Mohseni, B. Lägel, F. Ciubotaru, C. Adelmann, C. Dubs, S. D. Cotofana, O. V. Dobrovolskiy, T. Brächer, P. Pirro, A. V. Chumak, A magnonic directional coupler for integrated magnonic half-adders. *Nat. Electron.* **3**, 765–774 (2020).

18. T. Goto, T. Yoshimoto, B. Iwamoto, K. Shimada, C. A. Ross, K. Sekiguchi, A. B. Granovsky, Y. Nakamura, H. Uchida, M. Inoue, Three port logic gate using forward volume spin wave interference in a thin yttrium iron garnet film. *Sci. Rep.* **9**, 16472 (2019).

19. F. Schulz, F. Groß, J. Förster, S. Mayr, M. Weigand, E. Goering, J. Gräfe, G. Schütz, S. Wintz, Realization of a magnonic analog adder with frequency-division multiplexing. *AIP Adv.* **13**, 015115 (2023).

20. A. A. Nikitin, A. B. Ustinov, A. A. Semenov, A. V. Chumak, A. A. Serga, V. I. Vasyuchka, E. Lähderanta, B. A. Kalinikos, B. Hillebrands, A spin-wave logic gate based on a width-modulated dynamic magnonic crystal. *Appl. Phys. Lett.* **106**, 102405 (2015).

21. P. Chen, H. Wang, C. Cheng, C. Wan, D. Zhang, Y. Wang, Y. Wang, W. He, B. Chi, Y. Liu, G. Yu, H. Yu, X. Han, Complementary magnon transistors by comb-shaped gating currents. *Phys. Rev. Appl.* **20**, 054019 (2023).

22. A. Litvinenko, R. Khymyn, V. H. González, R. Ovcharov, A. A. Awad, V. Tyberkevych, A. Slavin, J. Åkerman, A spinwave Ising machine. *Commun. Phys.* **6**, 227 (2023).

23. A. N. Mahmoud, F. Vanderveken, F. Ciubotaru, C. Adelmann, S. Hamdioui, S. Cotofana, A spin wave-based approximate 4:2 compressor: Seeking the most energy-efficient digital computing paradigm. *IEEE Nanotechnol. Mag.* **16**, 47–56 (2022).

24. M. Balynsky, Y. Khivintsev, A. Kozhevnikov, Y. Nikulin, V. Sakharov, Y. Filimonov, A. Khitun, Magnonic active ring co-processor. *J. Appl. Phys.* **133**, 023904 (2023).

25. A. Mahmoud, F. Ciubotaru, F. Vanderveken, A. V. Chumak, S. Hamdioui, C. Adelmann, S. Cotofana, Introduction to spin wave computing. *J. Appl. Phys.* **128**, 161101 (2020).

26. U. Garlando, Q. Wang, O. V. Dobrovolskiy, A. V. Chumak, F. Riente, Numerical model for 32-bit magnonic ripple carry adder. *IEEE Trans. Emerg. Top. Comput.* **11**, 679–688 (2023).

27. Q. Wang, A. V. Chumak, P. Pirro, Inverse-design magnonic devices. *Nat. Commun.* **12**, 2636 (2021).

28. A. Papp, W. Porod, G. Csaba, Nanoscale neural network using non-linear spin-wave interference. *Nat. Commun.* **12**, 6422 (2021).

29. M. Kiechle, L. Maucha, V. Ahrens, C. Dubs, W. Porod, G. Csaba, M. Becherer, A. Papp, Experimental demonstration of a spin-wave lens designed with machine learning. *IEEE Magn. Lett.* **13**, 1–5 (2022).

30. B. Neşeli, Y. A. Yilmaz, H. Kurt, M. Turduev, Inverse design of ultra-compact photonic gates for all-optical logic operations. *J. Phys. D Appl. Phys.* **55**, 215107 (2022).

31. H. Wang, H. Xu, H. Huang, N. Zhou, H. Zhang, J. Li, Ultra-broadband and ultra-compact chip-integrated logic gates based on an inverse design method. *Opt. Laser Technol.* **169**, 110192 (2024).

32. Y. Lan, D. Li, H. Kai, B. Zou, G. Zhang, S. Feng, Inverse design of ultra-compact optical logic gates by genetic algorithm. *Opt. Commun.* **569**, 130800 (2024).

33. N. Zenbaa, C. Abert, F. Majcen, M. Kerber, R. O. Serha, S. Knauer, Q. Wang, T. Schrefl, D. Suess, A. V. Chumak, A universal inverse-design magnonic device. *Nat. Electron.* **8**, 106–115 (2025).

34. Q. Wang, R. Verba, B. Heinz, M. Schneider, O. Wojewoda, K. Davídková, K. Levchenko, C. Dubs, N. J. Mauser, M. Urbánek, P. Pirro, A. V. Chumak, Deeply nonlinear excitation of self-normalized short spin waves. *Sci. Adv.* **9**, eadg4609 (2023).

35. A. Khitun, Multi-frequency magnonic logic circuits for parallel data processing. *J. Appl. Phys.* **111**, 054307 (2012).

36. Q. Wang, R. Verba, K. Davídková, B. Heinz, S. Tian, Y. Rao, M. Guo, X. Guo, C. Dubs, P. Pirro, A. V. Chumak, All-magnonic repeater based on bistability. *Nat. Commun.* **15**, 7577 (2024).

37. A. Imre, G. Csaba, L. Ji, A. Orlov, G. H. Bernstein, W. Porod, Majority logic gate for magnetic quantum-dot cellular automata. *Science* **311**, 205–208 (2006).

38. F. Kronast, N. Friedenberger, K. Ollefs, S. Gliga, L. Tati-Bismaths, R. Thies, A. Ney, R. Weber, C. Hassel, F. M. Römer, A. V. Trunova, C. Wirtz, R. Hertel, H. A. Dürr, M. Farle, Element-specific magnetic hysteresis of individual 18 nm Fe nanocubes. *Nano Lett.* **11**, 1710–1715 (2011).

39. A. Haldar, D. Kumar, A. O. Adeyeye, A reconfigurable waveguide for energy-efficient transmission and local manipulation of information in a nanomagnetic device. *Nat. Nanotechnol.* **11**, 437–443 (2016).

40. C. Dubs, O. Surzhenko, R. Linke, A. Danilewsky, U. Brückner, J. Dellith, Sub-micrometer yttrium iron garnet LPE films with low ferromagnetic resonance losses. *J. Phys. D Appl. Phys.* **50**, 204005 (2017).

41. A. A. Serga, A. V. Chumak, B. Hillebrands, YIG magnonics. *J. Phys. D Appl. Phys.* **43**, 264002 (2010).

42. B. Shen, P. Wang, R. Polson, R. Menon, An integrated-nanophotonics polarization beamsplitter with $2.4 \times 2.4\ \mu m^2$ footprint. *Nat. Photonics* **9**, 378–382 (2015).

43. V. Bobkov, I. Zavislyak, V. Zagorodny, V. Romanjuk, “Microwave filters and multichannel divider based on surface magnetostatic waves,” in *12th International Conference Microwave and Telecommunication Technology* (IEEE, 2002), pp. 401–402.

44. K. Wu, A. Mortazawi, Nonreciprocal tunable low-loss bandpass filters with ultra-wideband isolation based on magnetostatic surface wave. *IEEE Trans. Microw. Theory Tech.* **60**, 3959–3968 (2012).
